# Nickel chloride (NiCl2) in hepatic toxicity: apoptosis, G2/M cell cycle arrest and inflammatory response

**DOI:** 10.18632/aging.101108

**Published:** 2016-11-05

**Authors:** Hongrui Guo, Hengmin Cui, Jing Fang, Zhicai Zuo, Junliang Deng, Xun Wang, Ling Zhao, Kejie Chen, Jie Deng

**Affiliations:** ^1^ College of Veterinary Medicine, Sichuan Agricultural University Ya'an 625014, China; ^2^ Key Laboratory of Animal Diseases and Environmental Hazards of Sichuan Province, Sichuan Agricultural University Ya'an 625014, China

**Keywords:** NiCl2, apoptosis, cell cycle G2/M phase arrest, inflammatory response, liver

## Abstract

Up to now, the precise mechanism of Ni toxicology is still indistinct. Our aim was to test the apoptosis, cell cycle arrest and inflammatory response mechanism induced by NiCl_2_ in the liver of broiler chickens. NiCl_2_ significantly increased hepatic apoptosis. NiCl_2_ activated mitochondria-mediated apoptotic pathway by decreasing Bcl-2, Bcl-xL, Mcl-1, and increasing Bax, Bak, caspase-3, caspase-9 and PARP mRNA expression. In the Fas-mediated apoptotic pathway, mRNA expression levels of Fas, FasL, caspase-8 were increased. Also, NiCl_2_ induced ER stress apoptotic pathway by increasing GRP78 and GRP94 mRNA expressions. The ER stress was activated through PERK, IRE1 and ATF6 pathways, which were characterized by increasing eIF2α, ATF4, IRE1, XBP1 and ATF6 mRNA expressions. And, NiCl_2_ arrested G_2_/M phase cell cycle by increasing p53, p21 and decreasing cdc2, cyclin B mRNA expressions. Simultaneously, NiCl_2_ increased TNF-α, IL-1β, IL-6, IL-8 mRNA expressions through NF-κB activation. In conclusion, NiCl_2_ induces apoptosis through mitochondria, Fas and ER stress-mediated apoptotic pathways and causes cell cycle G_2_/M phase arrest via p53-dependent pathway and generates inflammatory response by activating NF-κB pathway.

## INTRODUCTION

Ni is considered to be an essential element in microorganisms, plants, and animals, and has been a constituent of enzymes and proteins [1]. Due to its chemical properties, gloss, and low price, Ni has a wide variety of industrial applications [1]. Exposure to Ni has largely increased in industrial societies due to the environmental pollution by heavy metals at all stages of production, use, and disposal [2]. Human and animals contact Ni via several different pathways: ingestion, dermal contact, and inhalation [3]. After entering the body, Ni penetrates all organs and accumulates in various tissues and induces the tissue damage [4]. Ni can cause allergy, contact dermatitis, and toxicity of organ systems [4]. Ni may cause neurotoxicity, hepato-toxicity, nephrotoxicity, gene toxicity, reproductive toxicity, and increased risk of cancer [1, 4].

It has been reported that NiSO_4_ induces DNA damage, apoptosis and oxidative damage in the liver and testes of mouse and the liver of *Carassius auratus* [5–7]. Our studies have also shown that dietary NiCl_2_ in excess of 300 mg/kg can cause histopathological lesions, immunotoxicity, oxidative damage, apoptosis and cell cycle arrest in the kidney, thymus, spleen, small intestine, cecal tonsil and bursa of Fabricius of broiler chickens [8–22]. Also, previous research have demonstrated that Ni can induce apoptosis in B cells [23], human T hybridoma cells [24], HEp-2 and MCF-7 cells [25], CHO cells [26], and normal rat kidney cells [27]. So far, the extrinsic and intrinsic apoptotic pathways are widely explored in the apoptosis [28]. The intrinsic signaling pathways are known as mitochondria- and ER-initiated apoptosis [29, 30]. The extrinsic apoptotic pathway is activated by the binding of extracellular death ligands to cell-surface death receptors, and Fas-mediated apoptosis is the main pathway [31]. These references have demonstrated that Ni and Ni compounds induce apoptosis through mitochondria-mediated apoptosis, which are companied with the disruption of MMP, increase in cyt c and AIF translocation from mitochondria to cytoplasm and activation of caspase-9 and caspase-3 [25, 32, 33]. The results of Zhao et al. [33] suggest that metallic nickel particles can induce Fas-mediated apoptosis in JB6 cells. Recent studies have shown that the ER also plays an important role in the intrinsic pathway [34, 35]. Up to now, there is only a report that nickel acetate can induce ER stress and increase CHOP protein expression in NRK52E cells and Hepa-1c1c7 Cells [36]. It has also been reported that nickel acetate can induce G_2_/M phase arrest in Chinese hamster ovary cells [26], but the mechanisms is still unclear. Inflammatory response also induced by Ni and Ni compounds [37, 38]. Li et al. [39] have reported that NiCl_2_ increases the secretion of a pro-inflammatory cytokine (IL-1β) in bone marrow-derived macrophages and bone marrow dendritic cells. NiCl_2_ (20uM, 72h) increases pro-inflammatory cytokines (TNF-α, IL-β, and ICAM-1) mRNA expression levels in A549 cells [40].

Previous studies have suggested that Ni can induce apoptosis, cell cycle arrest and inflammatory response, however, the precise mechanism is still indistinct. There are no investigations on the Ni- or Ni compounds-induced Fas-mediated apoptotic pathway *in vitro*. Limited studies show the mechanism of NiCl_2_-induce ER stress, cell cycle arrest and inflammatory response at present. Therefore, the objective of this study was to determine potential mechanisms of NiCl_2_-induced apoptosis, cell cycle arest and inflammatory response. We measured the gene expression which involved in the apoptosis pathway, cell cycle G_2_/M phase and inflammatory response.

## RESULTS

### Clinical observation

The clinical observation was shown in the reference [22]. From 14 to 42 days of age during the experiment, broiler feed intake in the three NiCl_2_-treated groups began to decline when compared with those in the control group, except the 300 mg/kg group at 14 days of age. From 21 to 42 days of age during the experiment, broilers in the three NiCl_2_-treated groups showed inappetence, growth retardation and depression. A few broilers showed polypnea. No unexpected deaths occurred during the experiment.

### Histopathological changes in the liver

**Figure 1:**
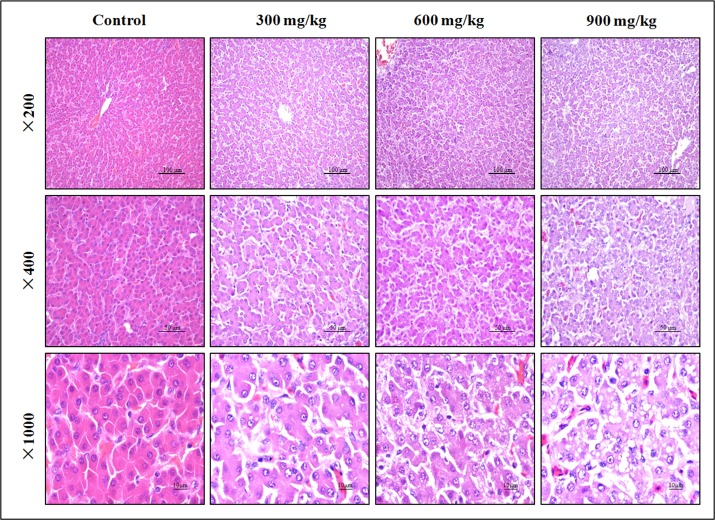
Histopathological changes in the liver at 42 days of age Control group: No changes are observed; 300 mg/kg group: Hepatic cells show slight granular and vacuolar degeneration; 600 mg/kg group: Hepatic cells show granular, vacuolar and fatty degeneration. 900 mg/kg group: Hepatic cells show marked granular, vacuolar and fatty degeneration. (H·E).

In Figures 1, NiCl_2_ resulted in dose-dependent histopathological changes in the liver, including granular degeneration, vacuolar degeneration, and fatty degeneration.

### Changes of hepatic function parameters

The activities of serum AST and ALT can directly reflect the damage of hepatic function. The results showed that AST and ALT activities were significantly higher (P < 0.05 or P < 0.01) in the 900 mg/kg group at 14 days of age and in the 600 and 900 mg/kg groups at 28 days of age and in the three NiCl_2_-treated groups at 42 days of age than those in the control group. ALP activity was higher (P < 0.05 or P < 0.01) in the 300, 600 and 900mg/kg groups than that in the control group from 14 to 42 days of age. GGT activity was also increased (P < 0.05 or P < 0.01) in the 600 and 900mg/kg groups at 28 and 42 days of age.

ALB, GLB and TP contents were lower (P < 0.05 or P < 0.01) in the 600 and 900mg/kg groups than those in the control group at 28 and 42 days of age. Also, TP content was decreased (P<0.05) in the 900mg/kg group at 14 days of age. TBIL content was increased (P < 0.05 or P < 0.01) in the 600 and 900mg/kg groups at 14 days of age and in the 300, 600 and 900mg/kg groups from 28 to 42 days of age when compared with that in the control group.

**Figure 2:**
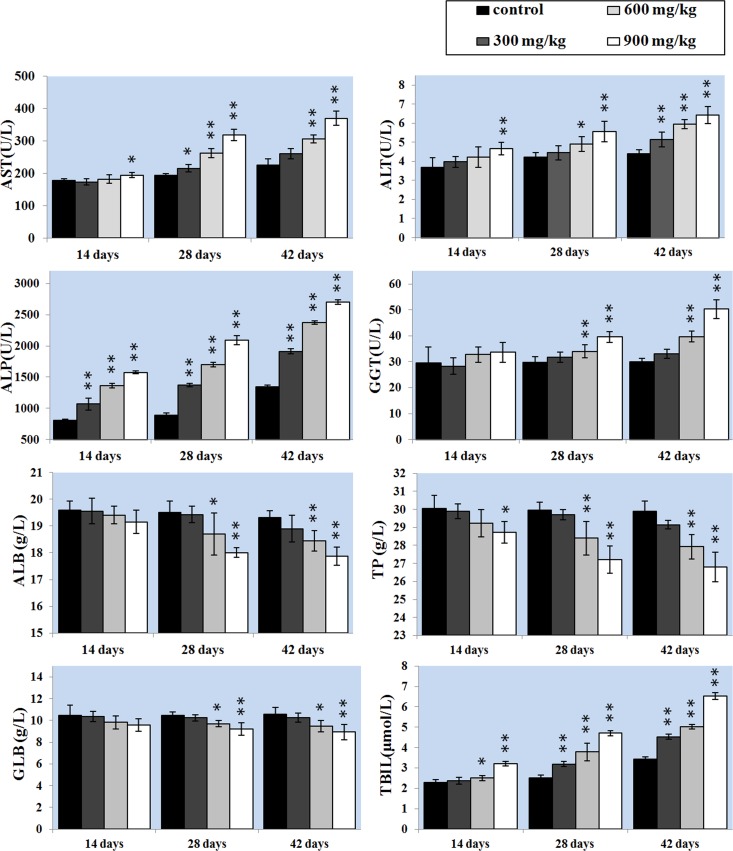
Changes of the hepatic function parameters Data are presented with the mean ± standard deviation (n=5). *P<0.05, compared with the control group. **P<0.01, compared with the control group.

The results were shown in Figure 2.

### Effects of NiCl_2_ on apoptosis in the liver

**Figure 3:**
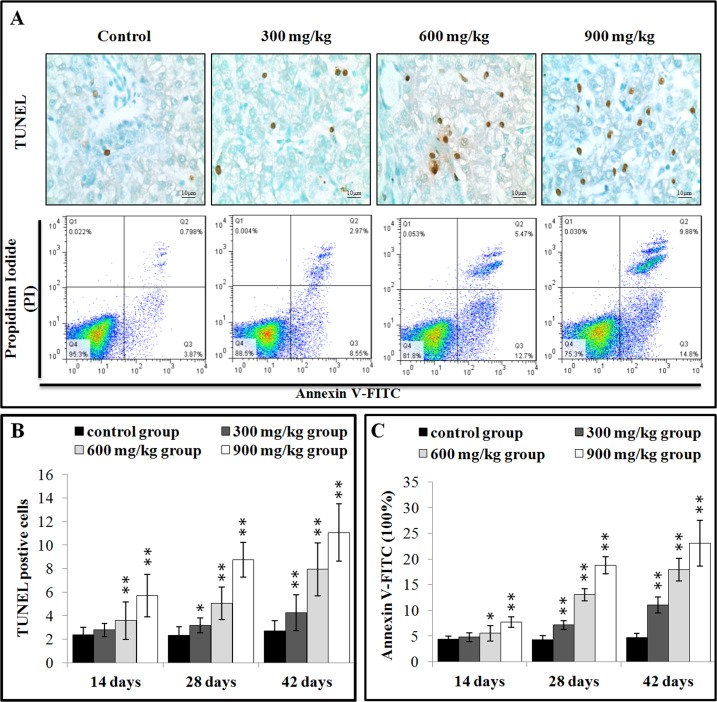
NiCl2 induces apoptosis in the liver (**A**) Representative TUNEL and flow cytometric diagram of apoptosis analysis. (**B**) Changes of the TUNEL positive cells in the liver. (**C**) Changes of the percentages of annexin V positive cells in the liver. Data are presented with the mean ± standard deviation B: (n=5×5), C: (n=5). *P<0.05, compared with the control group. **P<0.01, compared with the control group.

The effects of dietary NiCl_2_ on the apoptosis in the liver were observed with methods of the TUNEL and flow cytometry assay. The results presented in Figure 3B showed that the number of apoptotic cells was significantly greater (P < 0.05 or P < 0.01) in the 600 and 900 mg/kg groups at 14 days of age than in the control group. Apoptotic cells were significantly increased (P < 0.05 or P < 0.01) also in the three NiCl_2_-treated groups from 28 to 42 days of age.

After treated with NiCl_2_, hepatic cells were labeled using Annexin V-FITC and propidium iodide (PI) to discriminate live (Annexin V-FITC**2212** and PI**2212**), early apoptotic (Annexin V-FITC**002B** and PI**2212**), late apoptotic (Annexin V-FITC**002B** and PI**002B**) and primary/ secondary necrotic cells (Annexin V-FITC^¬^and PI**002B**). The results presented in Figure 3C showed that the apoptotic cells (early apoptotic + late apoptotic cells) were significantly higher (P < 0.05 or P < 0.01) in the 600 mg/kg and 900 mg/kg groups at 14 days of age, and in the three NiCl_2_-treated groups from 28 to 42 days of age than those in the control group.

### Effects of NiCl_2_ on apoptotic gene in the liver

**Figure 4:**
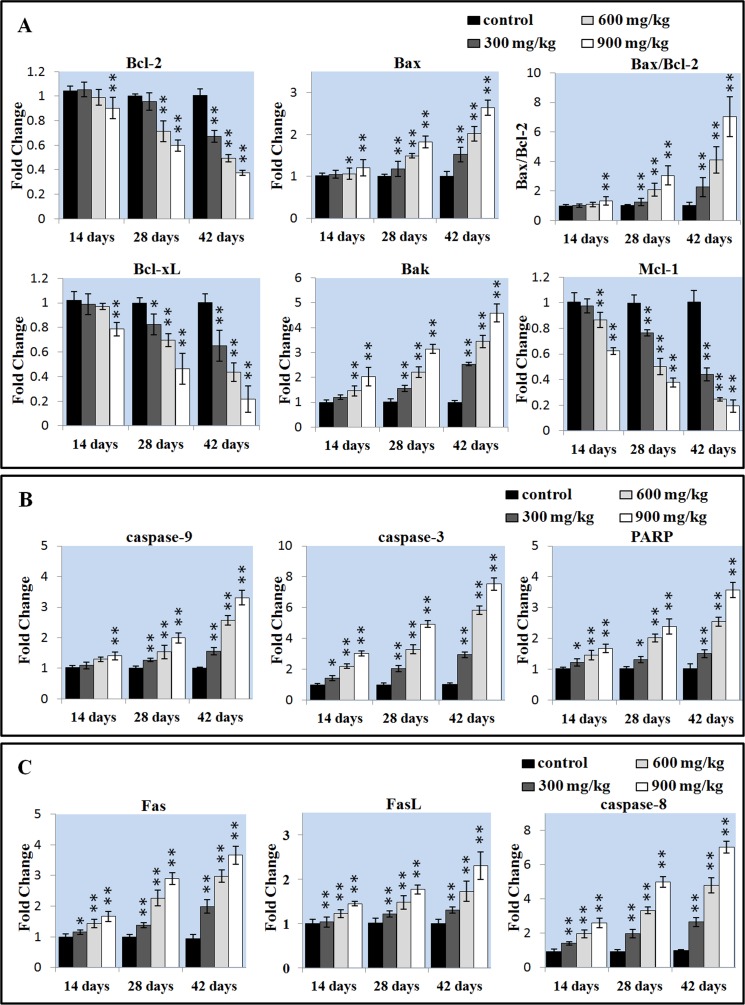
Changes of the apoptotic gene mRNA expression levels in the liver (**A**) Changes of the Bcl-2 family proteins mRNA expression levels. (**B**) Changes of the caspase-9, caspase-3 and PARP mRNA expression levels. (**C**) Changes of the Fas, FasL, caspase-8 proteins mRNA expression levels. Data are presented with the mean ± standard deviation (n=5). *P<0.05, compared with the control group. **P<0.01, compared with the control group.

The Bcl-2 family proteins have been shown to regulate the MMP. Therefore, the mRNA expressions of Bcl-2, Bax, Bcl-xL, Bak and Mcl-1 were detected and then the mRNA expression ratios of Bax/Bcl-2 were calculated. In Figure 4A, Bcl-2 mRNA expression was significantly decreased (P < 0.05 or P < 0.01) in the 900 mg/kg group at 14 days of age and in the 600 and 900 mg/kg groups at 28 days of age, and in the three NiCl_2_-treated groups at 42 days of age. Bax mRNA expression was significantly increased (P < 0.05 or P < 0.01) in the three NiCl_2_-treated groups from 28 to 42 days of age and in the 600 and 900 mg/kg groups at 14 days of age. Also, the Bax/Bcl-2 ratio was significantly higher (P < 0.05 or P < 0.01) in the three NiCl_2_-treated groups from 28 to 42 days of age and in the 900 mg/kg groups from 14 days of age than those in the control group. Bcl-xL and Mcl-1 mRNA expressions were significantly lower (P < 0.05 or P < 0.01) in the three NiCl_2_-treated groups from 28 to 42 days of age and in the 900 mg/kg group at 14 days of age than that in the control group. The mRNA expression of Bak was significantly increased (P < 0.05 or P < 0.01) in the three NiCl_2_-treated groups from 28 to 42 days of age and in the 600 and 900 mg/kg groups at 14 days of age.

The disruption of MMP results in the release of apoptosis-inducing protein cytochrome c (cyt-c) that promotes caspase-9, caspase-3 and PARP activation and apoptosis [41]. We detected the mRNA expression of caspase-3, caspase-9 and PARP in the liver. The caspase-9 mRNA expression was significantly increased (P < 0.05 or P < 0.01) in the three NiCl_2_-treated groups from 28 to 42 days of age and in the 900 mg/kg groups at 14 days of age when compared with those in the control group. The mRNA expressions of caspase-3 and PARP was significantly higher (P < 0.05 or P < 0.01) in the three NiCl_2_-treated groups from 14 to 42 days of age than that in the control group, as shown in Figure 4B.

We investigated whether Fas-mediated apoptosis pathway was involved in NiCl_2_-induced mediated apoptosis. As shown in Figure 4C, the Fas, FasL and caspase-8 mRNA expressions were significantly increased (P < 0.05 or P < 0.01) in three NiCl_2_-treated groups from 14 to 42 days of age when compared with those in the control group.

We also detected whether ER stress-mediated apoptosis pathway was involved in NiCl_2_-induced mediated apoptosis. We first examined whether NiCl_2_ could induce ER stress in the kidney. The extended ER stress can induce apoptosis. The results showed that NiCl_2_ significantly increased the mRNA expression of GRP78 and GRP94, which were the markers of ER stress. The GRP78 and GRP94 mRNA expression was significantly higher (P < 0.05 or P < 0.01) in the 600 and 900 mg/kg groups at 14 days of age and in the three NiCl_2_-treated groups from 28 to 42 days of age than that in the control group.

To further confirm that UPR pathways were involved in NiCl_2_-induced ER stress, we examined all the three UPR pathways: PERK pathway, IRE1 pathway and ATF6 pathway. The results indicated that NiCl_2_ induced the ER stress through activating PERK, IRE1 and ATF6 UPR pathways. In the PERK pathway, the eIF2α and ATF4 mRNA expressions were significantly higher (P < 0.05 or P < 0.01) in the three NiCl_2_-treated groups from 28 to 42 days of age than those in the control group. In the IRE1 pathway, the IRE1 mRNA expression was significantly increased (P < 0.05 or P < 0.01) in the 900 groups at 14 days of age, and in the 600 and 900 groups from 28 to 42 days of age when compared with that in the control group, and the XBP1 mRNA expression was significantly increased (P < 0.05 or P < 0.01) in the 900 groups at 14 days of age, in the 600 and 900 groups at 28 days of age, and in the three NiCl_2_-treated groups at 42 days of age when compared with that in the control group. In the ATF6 pathway, the ATF6 mRNA expression was significantly higher (P < 0.05 or P < 0.01) in the 600 and 900 mg/kg groups at 42 days of age than that in the control group.

### Effects of NiCl_2_ on cell cycle in the liver

Cell cycle includes S (DNA replication), M (nuclear division and cell division), G_1_ (the cell-cycle gap phase between M phase and S phase), G_2_ (the cell-cycle gap phase between S phase and M phase) phases, which is central to maintain homeostasis in the multicellular organisms [42]. We measured the percentage of G_0_/G_1_ phase (a prolonged nondividing state), S phase (DNA replication) and G_2_/M phase (the completed of DNA replication) cells in the liver.

**Figure 5:**
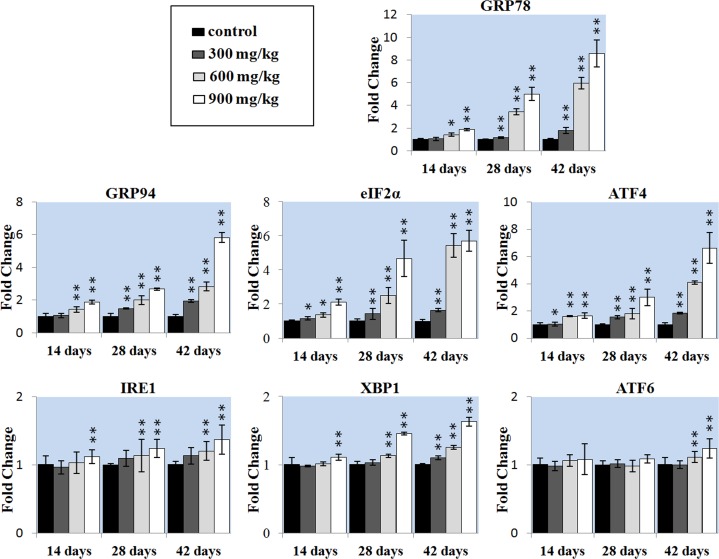
Changes of the ER stress gene mRNA expression levels in the liver Data are presented with the mean ± standard deviation (n=5). *P<0.05, compared with the control group. **P<0.01, compared with the control group.

As shown in Figure 5A and B, NiCl_2_ induced a dose-and time-dependent increase in G_2_/M phase cells and a corresponding decrease in cells at other stages of the cell cycle. The cell percentages in G_0_/G_1_ phase were significantly decreased (P < 0.05 or P < 0.01) in the 600 and 900 mg/kg groups at 28 days of age and in the three NiCl_2_-treated groups at 42 days of age when compared with those in the control group. The cell percentages in G_2_/M phase were significantly increased (P < 0.05 or P < 0.01) in the 900 mg/kg groups at 14 days of age and in the three NiCl_2_-treated groups from 28 to 42 days of age in comparison with those in the control group.

The mRNA expression changes of G_2_/M cell cycle regulatory molecule protein p53, p21, cdc2 and cyclin B were shown in Figures 5C. The p53 mRNA expression was significantly higher (P < 0.05 or P < 0.01) in the three NiCl_2_-treated groups from 14 to 42 days of age than that in the control group. The p21 mRNA expression significantly increased (P < 0.05 or P < 0.01) in the 900 mg/kg groups at 14 days of age and in the three NiCl_2_-treated groups from 28 to 42 days of age in comparison with those in the control group. The cdc2 mRNA expression was significantly lower (P < 0.05 or P < 0.01) in the 900 mg/kg group at 14 days of age, in the 600 and 900 mg/kg groups at 28 days of age and in the three NiCl_2_-treated groups at 42 days of age than that in the control group. The cyclin B mRNA expression was significantly decreased (P < 0.05 or P < 0.01) in the 600 and 900 mg/kg groups at 14 days of age and in the three NiCl_2_-treated groups from 28 to 42 days of age when compared with that in the control group.

### Effects of NiCl_2_ on inflammatory response in the liver

**Figure 6:**
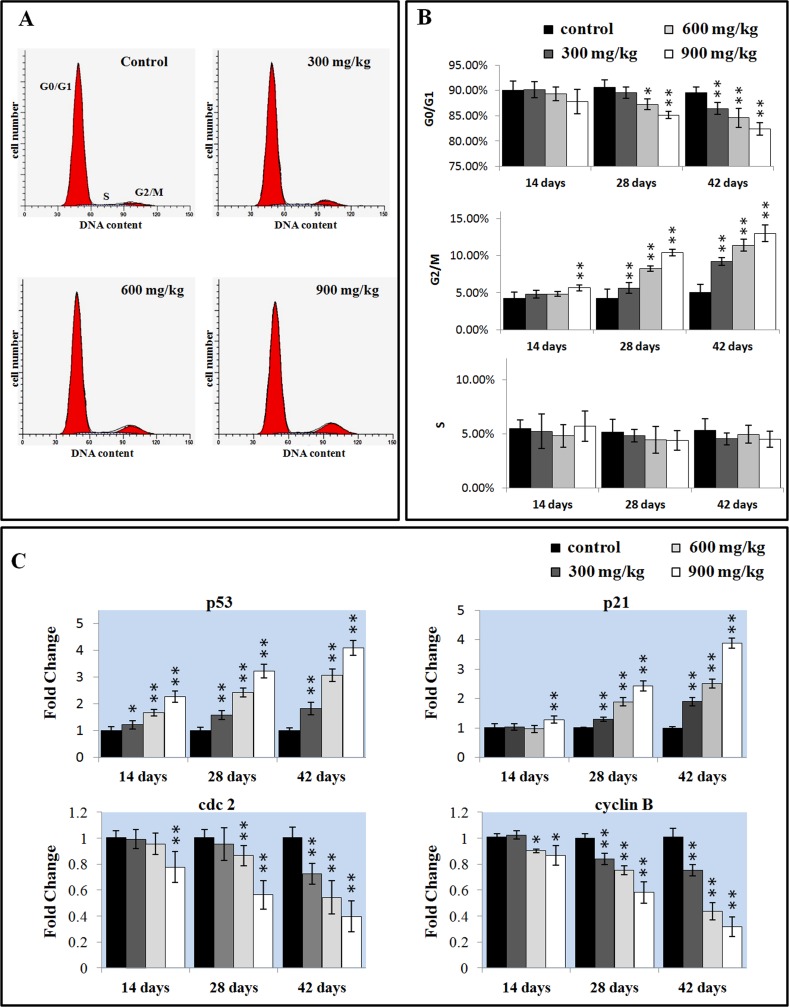
Changes of the cell cycle in the liver (**A**) Representative flow cytometric diagram of cell cycle analysis. (**B**) Changes of the percentage of G_0_/G_1_, G_2_/M, and S phase in the liver. (**C**) Changes of the G_2_/M phase regulators mRNA expression levels. Data are presented with the mean ± standard deviation (n=5). *P<0.05, compared with the control group. **P<0.01, compared with the control group.

As shown in Figure 6, the mRNA expressions of pro-inflammatory genes (NF-κB, TNF-α, IL-1β, IL-6 and IL-8) were significantly increased (P < 0.01) in the three NiCl_2_-treated groups from 28 to 42 days of age when compared with those in the control group. The TNF-α mRNA expression was significantly higher (P < 0.05 or P < 0.01) in the 600 and 900 mg/kg groups at 14 days of age than those in the control group. The IL-6 mRNA expression was significantly higher (P < 0.05 or P < 0.01) in the three NiCl_2_-treated groups at 14 days of age than those in the control group. The IL-8 mRNA expression was significantly increased (P < 0.01) in the 900 mg/kg groups at 14 days of age when compared with that in the control group.

**Figure 7:**
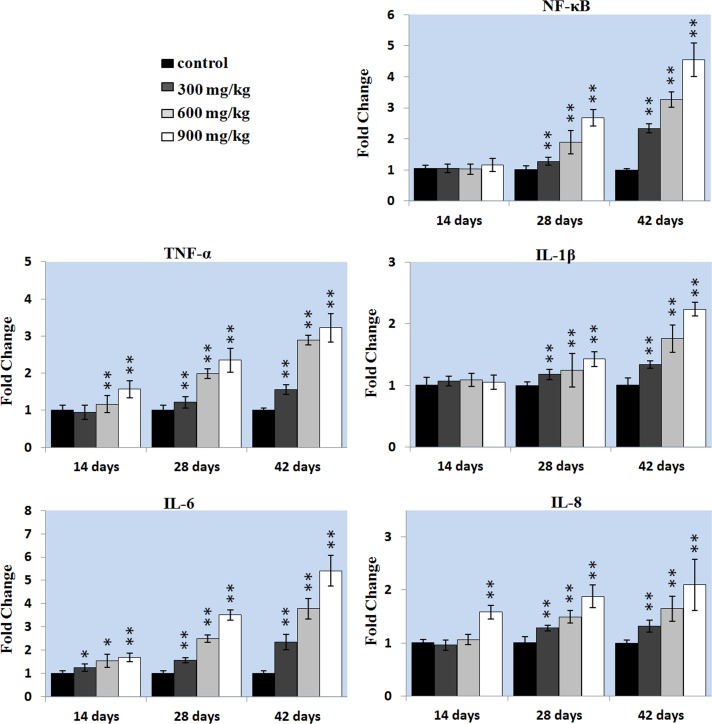
Changes of the inflammatory mediators mRNA expression levels in the liver Data are presented with the mean ± standard deviation (n=5). *P<0.05, compared with the control group. **P<0.01, compared with the control group.

### Changes of Ni contents in the liver

**Figure 8:**
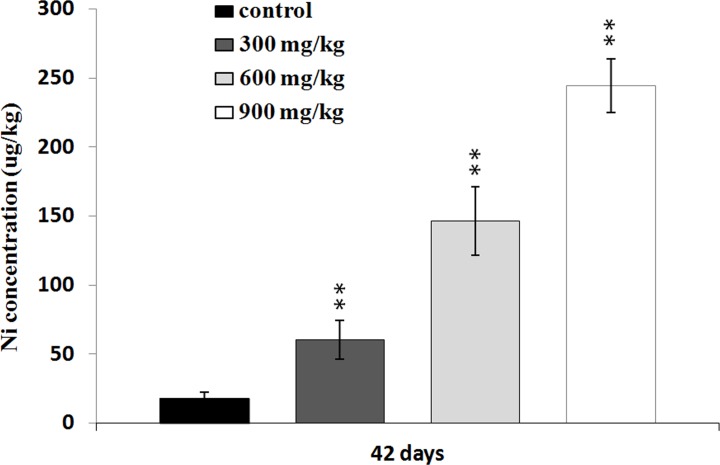
Changes of the Ni concentrations in the liver at 42 days Data are presented with the mean ± standard deviation (n=5). *P<0.05, compared with the control group. **P<0.01, compared with the control group.

The Ni contents were increased in the three NiCl_2_-treated groups at 42 days of age in comparison with those in the control group (P < 0.05 or P < 0.01), as shown in Figure 8.

## DISCUSSION

The liver is a primary site for xenobiotic metabolism and is the most common target organ for chemicals-induced injuries. Previous studies have shown that Ni exposure caused liver injuries and dysfunction [7, 43]. The present investigation, the decrease of serum ALB, GLB, TP contents and increase of serum AST, ALT, ALP, GGT activities and TBIL contents demonstrate that excessive NiCl_2_ induces hepatic damage. Moreover, histopathological changes of the liver also have been observed in NiCl_2_-treated broiler chickens. The histopathological injury and the alteration of hepatic function parameters are consistent with Ni accumulation in the liver, indicating that Ni accumulation is main or/and direct reason of the hepatic injury.

Many studies have suggested that one possible molecular mechanism involved in Ni toxicity is the excess apoptosis. Our previous studies have proved that NiCl_2_ induced apoptosis in the thymus, cecal tonsil, spleen, bursa of Fabricius, and kidney [8, 10, 17, 19, 44]. In this study, we found consistent evidence that dietary NiCl_2_ in excess of 300 mg/kg had adverse effects on the hepatic cells. The results of TUNEL and flow cytometry showed that NiCl_2_ significantly increased the percentage of apoptosis in the liver. In order to reveal the possible mechanism of NiCl_2_-induced apoptosis, we analyzed the mRNA expression of apoptotic genes which involved in mitochondria-, Fas- and ER stress-mediated apoptotic pathway. In the mitochondria-mediated apoptotic pathway, the balance between pro-apoptotic and anti-apoptotic Bcl-2 protein family members controls the mitochondrial apoptotic pathway [45]. The Bcl-2 family protein is consisting of apoptosis repressors (Bcl-2, Bcl-xL, and Mcl-1) and inducers (Bax and Bak) [45]. Loss of MMP is a prerequisite for mitochondrial-mediated apoptosis as it is associated with the reshuffling of Bcl-2 family proteins [46]. In this study, NiCl_2_ increased pro-apoptotic Bax and Bak mRNA expressions, and concomitantly decreased anti-apoptotic Bcl-2, Bcl-xL and Mcl-1 mRNA expressions. And, the Bax/Bcl-2 ratio was significantly increased. Our previous studies have also shown that NiCl_2_ increases Bax mRNA expression levels and decreases Bcl-2 mRNA expression levels in the thymus, cecal tonsil, kidney and spleen [8, 10, 17, 44]. Consistent with our findings, reduction of Bcl-2 and Bcl-xL protein expressions and enhancement of Bad, Bcl-Xs, Bax protein expressions have been observed after human proximal tubule cells and human bronchial epithelial cells have been cultured with nickel acetate and NiONPs [47, 48]. Ni_3_S_2_ down-regulates several anti-apoptotic proteins Bcl-2 and Bcl-xL in human BEAS-2B cells [49]. Changes of Bcl-2 family proteins disrupt the MMP, which translocates cyt-c from the mitochondrial intermembrane space to the cytosol [46]. Cyt-c can cleave and activate caspase-9, which then activate the downstream caspase-3. Caspase-3 is one of the key executioners of apoptosis, and is capable of cleaving or degrading many key proteins such as nuclear lamins, fodrin, and the nuclear enzyme PARP, which then induces apoptosis [50]. Our results showed that NiCl_2_ increased the mRNA expressions of caspase-3, caspase-9 and PARP, indicating that NiCl_2_ induced hepatic apoptosis through mitochondria-mediated apoptotic pathway. Our results are agreement with the results of Ahamed et al. [32] in which nickel ferrite nanoparticles increase activation and gene expression of caspase-3 and caspase-9 in HepG2 and MFC-7 cancer cells. An increase in the cleavage of procaspase-3 and PARP proteins has been also observed in nickel acetate-treated nasal epithelium [51].

In this study, the increased transcription levels of Fas, FasL, caspase-8 gene indicate the activation of Fas-mediated apoptotic pathway. In the Fas-mediated apoptotic pathway, FasL interacts with the Fas receptor, which then leads to caspase-8 activation [52]. Activated caspase-8 can directly cleave and activate caspase-3, which then causes apoptosis [52]. Zhao et al. [33] has also reported that nickel particles can increase Fas and caspase-8 expressions in JB6 cells.

Proper ER function is essential to cell survival, and perturbation of its function induces cellular damage and results in apoptosis [35]. Under prolonged and irreversible ER stress, cells that become irreversibly damaged are eliminated by apoptosis [35]. Our findings showed that NiCl_2_ induced ER stress, which was characterized by increasing ER stress markers GRP78 and GRP94 mRNA expressions. UPR is a defense mechanism against various cellular stress which causes accumulation of unfolded proteins in the ER [53]. In this study, NiCl_2_ increased transcription levels of eIF2a, ATF4, IREI, XBP1 and ATF6, implying that three UPR pathways: PERK pathway, IRE1 pathway and ATF6 pathway were involved in NiCl_2_-induced ER stress. Our findings are consistent with the results of our previous in the kidney [54]. PERK activates eIF2a, and then the phosphorylated eIF2a induces the translation of ATF4 mRNA [55]. IRE1 can activate XBP1 kinase and RNase activities to initiate XBP1 mRNA splice, which produces a potent transcriptional activator [56]. ATF6 is transported from ER to the golgi compartment, where it is cleaved to a cytosolic fragment that migrates to the nucleus to further activate the transcription of UPR genes [35]. Under prolonged ER stress, PERK pathway can trigger cell death. ATF4 can promote apoptosis through up-regulating the pro-apoptotic transcriptional factor CHOP [35]. CHOP induces the expression of several pro-apoptotic proteins such as GADD34, ERO1α and BH3-only proteins (BIM, PUMA and NOXA), thereby promoting apoptosis [57–59]. The IRE1 pathway has a pro-survival function, but it may cause apoptosis under prolonged ER stress [60]. IRE1 forms a complex with TRAF2 inducing the activation of ASK1/JNK, which triggers cell death [61, 62]. Furthermore, IRE1 also can activate NF-κB signaling that might lead to apoptosis [30].

The cell cycle analysis can give another insight into the toxicology mechanisms of NiCl_2_ in the liver. In the present study, a significant increase in the G_2_/M phase was observed in the liver of NiCl_2_-treated broiler chickens. A similar activation of G_2_/M phase arrest has been also observed in the nickel acetate-treated Chinese hamster ovary cells [26]. Our previous studies have suggested that NiCl_2_ induces G_2_/M phase arrest in the kidney, but G_0_/G_1_ phase arrest in the thymus and bursa of Fabricius [8, 18, 19]. The p53 protein is considered to be the most widespread inhibitor of cell proliferation, and p53-dependent G_2_/M cell cycle arrest is an important component of the cellular response to stress [63]. The cell cycle transition from G_2_ to M phase is strictly regulated by cdc2-cyclin B complex [64]. p53 can induce p21 up-regulation, which inhibits cdc2 activation, then causes cells to arrest in G_2_/M phase [65]. In this study, the results showed that the G_2_/M phase arrest process was associated with the increased p53 and p21mRNA expressions, and decreased cdc2 and cyclin B mRNA expressions. All of the present evidence suggests that NiCl_2_ may induce G_2_/M phase accumulation by inhibiting the cdc2-cyclinB complexes through directly stimulating the p53 and p21 expressions. Lee et al. [51] has demonstrated that nickel acetate-induced G_2_/M arrest is associated with up-regulation of p53, p21 expressions, decrease in phosphorylation of cdc2, and down-regulation of cyclin B1 expression in nasal epithelium. Salnikow et al. [66, 67] also has observed that NiCl_2_ and nickel sulfide (Ni_3_S_2_) up-regulate p53 protein levels in human lung cells, MCF-7 and A549 cells. The p53 protein not only leads to cell cycle arrest in the G_2_/M phase of the cell cycle by directly stimulating the p21expression, but also promotes apoptosis through up-regulation of Bax and down-regulation of Bcl-2 protein expression [63].

In this study, we also found that NiCl_2_ induced hepatic inflammatory responses, which is company with the up-regulation of pro-inflammatory genes (TNF-a, IL-1β, IL-6 and IL-8) mRNA expression levels and the increased NF-κB transcription level. The excessive mRNA expressions of proinflammatory cytokines further caused cell functional disorder and apoptosis [68]. NF-κB is highly activated at sites of inflammation in diverse diseases and can induce transcription of proinflammatory cytokines, chemokines, adhesion molecules [68]. Our results indicated that NiCl_2_ amplified proinflammatory mediators secretion by activating the NF-κB signaling pathway in this inflammatory response, which was in agreement with the results obtained by Freitas et al. [69]. Capasso et al. [38] also suggest that nickel oxide nanoparticles induce inflammation through activation of NF-κB pathway in lung epithelial cells.

In conclusion, dietary NiCl_2_ induces apoptosis through mitochondria, Fas, ER stress-mediated apoptotic pathway and causes cell cycle G_2_/M phase arrest via p53-dependent pathway and generates inflammatory response by NF-κB pathway activation. The prolonged cell cycle G_2_/M phase arrest and inflammatory response also can induce apoptosis.

## MATERIALS AND METHODS

### Experimental design

Two hundred and eighty one-day-old healthy broiler chickens (Chia Tai Group, Wenjiang, Sichuan, China) were divided into four groups (N = 70), and were housed in cages with electrical heaters and provided with feed and water as well as the under-mentioned experimental diets ad libitum for 42 days. The growth cycle of commercial broilers is about 42 days, after which they are used for consumption. In this rapid growth period food consumption is high, and broilers will easily be affected by diet containing metal pollutants (such as Ni). The aim of our study is to evaluate the effect of dietary NiCl_2_ on the broilers in this period of rapid growth.

To observe the time-dependent dynamic change, we chose three time points (14, 28, and 42 days of age) for examining hepatic function, the alterations of apoptosis, cell cycle and inflammatory response.

A corn-soybean basal diet formulated by the National Research Council [70] was the control diet, and NiCl_2_ (NiCl_2_·6H_2_O, Cheng Du Kelong Chemical Co., Ltd., Chengdu, China) was mixed into this basal diet to produce experimental diets containing 300, 600 and 900 mg/kg NiCl_2_, respectively.

The basis of doses (300, 600 and 900 mg/kg NiCl_2_) selection: Ling and Leach reported that dietary NiCl_2_ concentrations of 300 mg/kg and over resulted in significant reduction in growth rate. Mortality and anemia were observed in chicks receiving 1100 mg/kg nickel [71]. Weber and Reid found a significant growth reduction at 700 mg/kg NiSO_4_ and nickel acetate and over [72]. Chicks fed more than 250-300 mg/kg Ni in the diet exhibited depressed growth and reduced feed intake [73]. Bersenyi et al. [74] reported that supplementation of 500 mg/kg NiCl_2_ reduced weight gain (by 10%), feed intake (by 4%) and worse feed FCE (by 5%) in growing broiler cockerels. According to the above-mentioned research results and our preliminary experiment, we chose the doses of 300, 600 and 900 mg/kg NiCl_2_ in this study for observing the does-dependent changes.

The animal protocols and all procedures of the experiment were performed in compliance with the laws and guidelines of Animal Care and Use Committee, Sichuan Agricultural University (Approval No: 2012-024).

### Histopathological examination of liver

Five broiler chickens in each group were humanely killed at 42 days of age. Livers were removed, fixed in 4% paraformaldehyde, dehydrated in ethanol and embedded in paraffin. Serial slices at 5 μm thickness were prepared and stained with haematoxylin and eosin (H**·**E), and examined by light microscopy.

### Determination of the hepatic function parameters

At 14, 28, and 42 days during the experiment, five broiler chickens in each group were phlebotomized from jugular vein to collect serum. Non-anticoagulative blood samples were clotted for 15 min at room temperature and then centrifuged at 3000 rpm for 15 min. The serum ALT and AST, ALP, GGT and the contents of TP, ALB, GLB, TBIL were detected by biochemical methods following the instruction of the reagent kits (these kits purchased from Nanjing Jiancheng Bioengineering Institute of China, Nanjing, China).

### Apoptosis analysis by flow cytometry

At 14, 28, and 42 days of age, five broiler chickens in each group were taken for determination of the changes of apoptosis in the liver by flow cytometry.

The broiler chickens in each subsample were humanely killed, and their livers were immediately taken and ground to form a cell suspension, which was filtered through a 300-mesh nylon screen. The cells were washed twice with ice-cold phosphate buffer saline (PBS, pH 7.2-7.4), and then suspended in PBS at a concentration of 1×10^6^ cells/mL. A total of 100 μL of the cell suspension was transferred to a 5-mL culture tube. The cells were respectively stained with 5 μL Annexin V-FITC (Cat: 51-65874X, BD, USA) and 5 μL of PI (Cat: 51-66211E, BD, USA) at 25ºC in the dark. Finally, 400 μL of 1×binding buffer were added to each tube after 15 minutes, and cells were analyzed by flow cytometry (BD FACSCalibur) within 40 min of preparation. The results were analyzed using the Mod Fit LT for Mac V3.0 computer program.

### Detection of hepatic apoptosis by TUNEL

Five broiler chickens in each group were humanely sacrificed at 14, 28, and 42 days of age. Livers were removed, fixed in 4% paraformaldehyde, dehydrated in ethanol and embedded in paraffin.

TUNEL analysis was carried out according to the manual of In Situ Cell Death Detection Kit (Cat: 11684817980, Roche, Mannheim, Germany). Briefly, tissue slices (5 μm thick) were rehydrated in a series of xylene and ethanol solutions and then rinsed in ddH2O, digested with 50 μL proteinase K (diluted in Tris·HCl pH 7.8) for 15 min, and incubated with 3% H_2_O_2_ in methanol for 15 min at room temperature to inactivate endogenous peroxidase. The slices were transferred to a reaction mixture containing biotin-dUTP terminal deoxynucleotidyl and incubated in a humidified chamber for 1 h at 37°C, followed by washing in PBS (pH 7.2-7.4). Slices were incubated in Converter-POD (HRP) for 30 min at 37°C. Reaction product was visualized with DAB kit (AR1022, Boster, Wuhan, China). After final washing in ddH_2_O, slices were lightly counterstained with methyl green, dehydrated in ethanol, cleared in xylene and mounted.

Cells were observed with light microscopy (Olympus, Shimadzu, Japan). The nuclei of apoptotic cells containing DNA strand breaks were stained brown. The TUNEL positive cells (apoptotic cells) were counted by use of a computer-supported imaging system connected to a light microscope with an objective magnification of ×1000. Apoptotic cells were quantified by use of Image-Pro Plus 5.1 (Madia Cybernetics, Bethesda, MD, USA) image analysis software. Five slices in each chicken and five fields in each slice were measured and averaged.

### Cell cycle analysis by flow cytometry

At 14, 28, and 42 days of age, five broiler chickens in each group were taken for determination of the cell cycle stages in the liver by flow cytometry.

The broiler chickens in each subsample were humanely killed, and their livers were immediately taken and ground to form a cell suspension, which was filtered through a 300-mesh nylon screen. The cells were washed twice with ice-cold PBS (pH 7.2-7.4), and then suspended in PBS at a concentration of 1 × 10^6^ cells/mL. A total of 500 μL of the cell suspension was transferred to a 5-mL culture tube. After centrifugation (600 rpm, 5 min), the supernatant was decanted, the cells were incubated for 30 min at room temperature in the dark with 5 μL 0.25% Triton X-100 and 5 μL PI (Cat. No.51-66211E). Finally, 500 μL of PBS were added to each tube, and cells were analyzed by flow cytometry (BD FACSCalibur) within 45 min of preparation. The results were analyzed using the Mod Fit LT for Mac V3.0 computer program.

### Determination of the mRNA expression by quantitative real-time PCR

The livers from five broiler chickens in each group were taken at 14, 28, and 42 days of age and stored in liquid nitrogen. They were then homogenized in liquid nitrogen using a mortar and pestle. The total RNA was isolated using RNAiso Plus (9108/9109, Takara, Japan). Subsequently, RNA was transferred to cDNA using a Prim-Script™ RT reagent Kit (RR047A, Takara, Japan) according to the manufacture's protocol. The cDNA product was used as a template for qRT-PCR analysis. Sequences for target genes were obtained from the NCBI database. Oligonucleotide primers were designed using Primer 5 software and synthesized at Takara (Dalian, China; see Table 1, 2 and 3).

**Table 1:** A list of primers of the apoptotic genes in qRT-PCR analysis

Gene symbol	Accession number	Primer	Primer sequence(5′-3′)	Product size	Tm (°C)
Bcl-2	NM205339	Forward Reverse	GATGACCGAGTACCTGAACC CAGGAGAAATCGAACAAAGGC	114bp	61
Bax	XM422067	Forward Reverse	TCCTCATCGCCATGCTCAT CCTTGGTCTGGAAGCAGAAGA	169bp	62
Bcl-xL	GU230783	Forward Reverse	ATGAGTTTGAGCTGAGGTACCGG AGAAGAAAGCCACGATGCGC	150bp	59
Bak	NM001030920	Forward Reverse	TCTACCAGCAAGGCATCACGG ATCGAGTGCAGCCACCCATC	122bp	60
Mcl-1	XM001233734	Forward Reverse	GGATCATCACGGACGCATTG TGATGCTGCTTTCCAGGTCC	111bp	60
caspase-9	AY057940	Forward Reverse	CGAAGGAGCAAGCACGACAG CCGCAGCCCTCATCTAGCAT	130bp	61
caspase-3	NM204725	Forward Reverse	TGGCCCTCTTGAACTGAAAG TCCACTGTCTGCTTCAATACC	139bp	62
PARP	NM205263	Forward Reverse	AAGCTCCGAACTGATATTAAGGTGG GCTTAAATGGCTTGTAACGCTGA	172bp	56
Fas	NM001199487	Forward Reverse	TGTTCGTCATCACCGTCTATCG TTCGTAGGCTCCTCCCATCC	133bp	60
FasL	AJ890143	Forward Reverse	AGATCGCATCCCTCCAGCTC GAGACAGGTTCCCACTCCAATG	135bp	59
Caspase-8	NM204592	Forward Reverse	TGGGAAAGTGGACAAGAGCCT CCACAGATGATGCCAGCCAA	146bp	59
GRP78	NM205491	Forward Reverse	GAATCGGCTAACACCAGAGGA CGCATAGCTCTCCAGCTCATT	118bp	59
GRP94	NM204289	Forward Reverse	CTTCGCTTCCAGTCTTCCCATC AGAAGGCGTTCAACAAATGGTG	149bp	58
Eif2a	NM001031323	Forward Reverse	GCTGCGAGTCAGTAATGGGTATAA CTGCCAGGAAACTTGCCACA	103bp	59
ATF4	AB013138	Forward Reverse	TTGATGCCCTGTTAGGTATGGAA GGTATGAGTGGAGGTTCTTTGTTGT	139bp	60
IRE1	NM001285499	Forward Reverse	TGAGGGCAATGAGAAATAAGAAGC TGTAGGAGCAGGTGAGGGAAGC	127bp	61
XBP1	NM001006192	Forward Reverse	GCGAGTCTACGGATGTGAAGGA TGTGGAGGTTGTCAGGAATGGT	140bp	61
ATF6	XM422208	Forward Reverse	GATTGTGGGCGTCACTTCTCG TGGGATGCCAATGTTAGCCTG	142bp	57
β-actin	L08165	Forward Reverse	TGCTGTGTTCCCATCTATCG TTGGTGACAATACCGTGTTCA	178bp	62

**Table 2:** A list of primers of the cell cycle regulator genes in qRT-PCR analysis

Gene symbol	Accession number	Primer	Primer sequence(5′-3′)	Product size	Tm (°C)
p53	NM205264.1	Forward Reverse	ACCTGCACTTACTCCCCGGT TCTTATAGACGGCCACGGCG	127bp	59
p21	AF513031.1	Forward Reverse	TCCCTGCCCTGTACTGTCTAA GCGTGGGCTCTTCCTATACAT	123bp	60
cdc2	NM205314.1	Forward Reverse	TCTGCTCTGTATTCCACTCCTG ATTGTTGGGTGTCCCTAAAGC	144bp	60
cyclinB	NM205239.2	Forward Reverse	ATCACCAACGCTCACAAGAAC AGGCTCCACAGGAACATCTG	171bp	59
β-actin	L08165	Forward Reverse	TGCTGTGTTCCCATCTATCG TTGGTGACAATACCGTGTTCA	178bp	62

**Table 3:** A list of primers of the inflammatory mediator genes in qRT-PCR analysis

Gene symbol	Accession number	Primer	Primer sequence(5′-3′)	Product size	Tm (°C)
NF-κB	NM205134	Forward Reverse	CTGAAACTACTGATTGCTGCTGGA GCTATGTGAAGAGGCGTTGTGC	179bp	62
TNF-α	NM204267	Forward Reverse	CCCCTACCCTGTCCCACAA TGAGTACTGCGGAGGGTTCAT	100bp	58
IL-1β	Y15006	Forward Reverse	CAGCCTCAGCGAAGAGACCTT CACTGTGGTGTGCTCAGAATCC	106bp	60
IL-6	AJ309540	Forward Reverse	AATCCCTCCTCGCCAATCTG GCCCTCACGGTCTTCTCCATA	106bp	60
IL-8	HM179639	Forward Reverse	GCCCTCACGGTCTTCTCCATA CTGGCCCTCCTCCTGGTT	105bp	60
β-actin	L08165	Forward Reverse	TGCTGTGTTCCCATCTATCG TTGGTGACAATACCGTGTTCA	178bp	62

For qRT-PCR reactions, 25μL mixtures were made containing 12.5μL SYBR^®^ Premix Ex Taq^TM^ II systemII (DRR820A, Takara, Japan), 1μL of forward and 1μL reverse primer, 8.5μL of RNAase-free water (RT12102, Tiangen, China) and 1μL of cDNA. Bio Rad C1000 Thermal Cycler (Bio Rad, USA) was used to perform qRT-PCR reactions. The PCR procedure consisted of 95°C for 3 min followed by 44 cycles 95°C for10 s, Tm of a specific primer pair for 30 s, and then 95°C for 10 s, 72°C for 10 s. The melting curve analysis showed only one peak for each PCR product.

Chicken β-actin expression was used as an internal reference housekeeping gene. Gene expression values from control group subsamples at 14, 28, and 42 days of age were used to calibrate gene expression in subsamples from corresponding experimental subsamples. All Ddata output from the qRT-PCR experiments were analyzed using the 2^−ΔΔCT^ method [75].

### Determination of the hepatic Ni contents by GFAAS

After five broilers in each group were humanely killed at 42 days of age, livers were immediately removed, weigh-ed, dried, and collected for determination of Ni contents.

Ni concentrations in the liver were measured by GFAAS according to the reference [16].

### Statistical analysis

The significance of difference among four groups was analyzed by variance analysis, and results presented as mean ± standard deviation (M±SD). The variation was measured by one-way analysis of variance (ANOVA) test of SPSS 16.0 for windows. Statistical significance was considered at P < 0.05.

## Abbreviations

Ni: nickel
NiCl_2_: nickel chloride
NiSO_4_: nickel sulfate
HEp-2: human airway epithelial
MCF-7: human breast cancer
CHO: Chinese hamster ovary
ER: endoplasmic reticulum
MMP: mitochondrial membrane potential
cyt c: cytochrome c
AIF: apoptosis inducing factor
CHOP: C/EBP homologous protein
IL-1β: interleukin-1β
TNF-α: tumor necrosis factor α
ICAM-1: intercellular cell adhesion molecule-1
AST: aspartate aminotransferase
ALT: alanine aminotransferase
ALP: Alkaline phosphatase
GGT: Glutamyltransferase
ALB: albumin
GLB: globulin
TP: total protein
TBIL: total bilirubin
TUNEL: terminal deoxynucleotidyl transferase 2′-deoxyuridine 5′-triphosphate dUTP nick end-labeling
Bcl-xL: Bcl-extra long
Mcl-1: myeloid cell leukemia factor-1
PARP: poly ADP-ribose polymerase
FasL: Fas ligand
GRP78: glucose-regulated protein 78
UPR: unfolded protein response
PERK: protein kinase RNA (PKR)-like ER kinase
IRE1: inositol-requiring enzyme 1
ATF6: activated transcription factor 6
eIF2α: elongation initiation factor 2α
XBP1: X-boxbinding protein 1
NF-κB: nuclear factor-κB
NiONPs: NiO nanoparticles
Ni_3_S_2_: nickel sulfide
GADD34: growth arrest and DNA-damage-inducible 34
ERO1α: endoplasmic reticulum oxido-reductase 1α
TRAF2: TNF receptor-associated factor 2
ASK1/JNK: apoptosis signal-regulating kinase 1/c-Jun amino terminal kinase
FCE: feed conversion efficiency
PI: propidium iodide
GFAAS: graphite furnace atomic absorption spectrometry
